# Undetectable brain natriuretic peptide as a marker of ultrasound-detected hypovolemia in patients with cancer on active treatment

**DOI:** 10.3389/fcvm.2026.1606572

**Published:** 2026-01-27

**Authors:** Amy Tu Trinh Le, Raed Qarajeh, Bart Wilder, Akash Sharma, Lolita Golemi, Tochukwu Okwuosa

**Affiliations:** 1 Department of Internal Medicine, Rush University Medical College, Chicago, IL, United States; 2 Department of Internal Medicine, Division of Cardiology, Rush University Medical Center, Chicago, IL, United States; 3 Department of Internal Medicine, Rush University Medical Center, Chicago, IL, United States; 4 Department of Internal Medicine, University of Buffalo (Catholic Health System – Sisters of Charity Hospital), Buffalo, NY, United States; 5 Department of Internal Medicine, Saint Louis University Hospital, Saint Louis, MO, United States

**Keywords:** brain natriuretic peptide, cancer, inferior vena cava, transthoracic echocardiography, hypovolemia

## Abstract

Hypovolemia is a common complication in patients undergoing cancer-related therapies, yet assessing volume status in this population is challenging because of confounding comorbidities. This retrospective study is the first of its kind to examine the relationship between hypovolemia, assessed by measuring inferior vena cava (IVC) diameter via transthoracic echocardiogram (TTE) as an indicator of fluid status, and brain natriuretic peptide (BNP) levels, a cardiac neurohormone released by the ventricles in response to increased pressure. We hypothesized that undetectable BNP levels (<10 pg/mL) would associate with small IVC diameters indicative of hypovolemia. Although our study revealed wide confidence intervals reflective of a small sample size, our findings suggest that undetectable BNP, a readily available laboratory marker, may serve as an indicator of hypovolemia when physical examination findings are inconclusive. In addition, our study was limited by the 2-week temporal variability of BNP and IVC measurements, which may potentially introduce bias because of the dynamic fluctuation of volume status. Further studies with larger cohorts and closer temporal alignment of BNP and TTE measurements are needed to validate these findings.

## Introduction

Hypovolemia, a state of decreased intravascular fluid, is frequently seen in patients with cancer because of catabolism, cachexia, and decreased oral intake (1, 2). However, volume status assessment in this population is often difficult because of competing comorbid conditions. Brain natriuretic peptide (BNP), a cardiac neurohormone secreted by the stretched ventricles during increased pressure, is a diagnostic tool for heart failure and is affected by cancer-related treatments (3, 4). The size of the inferior vena cava (IVC) is a tool to assess volume status. A dilated IVC is indicative of hypervolemia and a collapsible IVC is indicative of hypovolemia (5). Using IVC diameter as a marker for fluid status, measured by a transthoracic echocardiogram (TTE), we, in this study, evaluated the relationship between hypovolemia and BNP levels in patients undergoing cancer-related therapy. We hypothesized that a small IVC diameter would correlate with BNP levels <10 pg/mL. We strictly adhered to the Strengthening the Reporting of Observational Studies in Epidemiology (STROBE) guidelines while conducting this retrospective study.

## Methods

A total of 89 patients (age range 18–80 years) with solid malignancies and currently undergoing chemo- or radiation therapy were categorized into 3 groups based on BNP values: undetectable (BNP <10 pg/mL), normal (BNP 10–100 pg/mL), and high (BNP >100 pg/mL). Patients with the following comorbidities likely to affect BNP levels were excluded from the analysis: renal failure, atrial fibrillation, uncontrolled hypertension [systolic blood pressure (BP) >140 mmHg in any of the last three clinic visits], pulmonary disease, age >80 years, and obesity (BMI >30 kg/m^2^). Retrospective data were obtained between 2016 and 2023 from the Rush Medical Center Cardio-Oncology Registry. IVC measurements were acquired by three blinded observers (two medical trainees and a board-certified reference cardiologist) from TTEs obtained within 2 weeks of BNP measurements. Small IVC was defined as maximal diameter less than 1.2 cm with a high degree of respiratory collapsibility according to the American Society of Echocardiography guidelines. Ordinal regression was used to calculate the adjusted odds ratio (OR) with a 95% confidence interval (CI) of the measured IVC diameters per data point. The difference in OR for IVC diameters was compared between the three BNP groups.

## Results

There were 26, 31, and 32 patients with median ages of 54.89, 59.06, and 65.22 years in the undetectable, normal, and high BNP groups, respectively. The Intraclass Correlation Coefficient among all observers was 0.805 (*p* < 0.001). A statistically significant stepwise categorical drop in BNP levels was associated with a categorically small IVC diameter (*p* = 0.003; Table 1). In the univariate analysis, a categorical (undetectable) BNP <10 pg/mL compared with normal or high BNP was significantly associated with a small IVC diameter suggestive of hypovolemia (*p* = 0.005; OR 3.90, 95% CI [1.55, 10.6]). This relationship held when adjusted for age in Model 1 (*p* = 0.01; OR 3.39, 95% CI [1.33, 9.27]), and age, sex, BMI, systolic BP, creatinine, and albumin in Model 2 (*p* = 0.001; OR 7.04, 95% CI [2.31, 24.67]) (Table 2).

**Table 1:** Baseline characteristics.

Variables	Overall *N* = 89	<10 *N* = 26	11–100 *N* = 31	>100 *N* = 32	*p*-value
Demographics^a^					
Age (mean ± SD)	59.99 ± 12.19	54.89 ± 13.88	59.06 ± 12.80	65.22 ± 7.37	0.004
Sex					0.79
Female (%)	61 (67%)	19 (70%)	22 (69%)	20 (62%)	
Male (%)	30 (33%)	8 (30%)	10 (31%)	12 (38%)	
BMI ± SD	30.44 ± 9.20	31.53 ± 8.67	29.73 ± 9.47	30.22 ± 9.56	0.75
Total number of comorbidities^a^					0.88
0 (%)	4 (4.4%)	2 (7.4%)	1 (3.1%)	1 (3.1%)	
1 (%)	29 (32%)	9 (33%)	10 (31%)	10 (31%)	
2 (%)	34 (37%)	10 (37%)	11 (34%)	13 (41%)	
3 (%)	18 (20%)	4 (15%)	9 (28%)	5 (16%)	
4 (%)	6 (6.6%)	2 (7.4%)	1 (3.1%)	3 (9.4%)	
Cancer characteristics^a^					
Number with lung cancer	15 (16%)	2 (7.4%)	9 (28%)	4 (12%)	
Number with breast cancer	28 (31%)	8 (30%)	11 (34%)	9 (28%)	
Number treated with chemo	91 (100%)	27 (100%)	32 (100%)	32 (100%)	
Number treated with anthracycline-based chemo	39 (45%)	12 (48%)	13 (43%)	14 (44%)	0.93
Number treated with radiation therapy	38 (42%)	9 (33%)	14 (44%)	15 (47%)	0.55
Laboratory parameters^a^					
Sodium (mmol/L, mean ± SD)	139.12 ± 3.05	139.59 ± 2.52	138.69 ± 3.48	139.16 ± 3.03	0.53
Albumin (g/dL, mean ± SD)	3.29 ± 0.59	3.60 ± 0.37	3.18 ± 0.70	3.09 ± 0.52	0.005
BUN (mg/dL, mean ± SD)	17.31 ± 10.40	12.54 ± 4.13	14.91 ± 4.90	23.75 ± 14.38	<0.001
Creatinine (mg/dL, mean ± SD)	1.05 ± 0.76	0.87 ± 0.30	0.88 ± 0.21	1.37 ± 1.18	0.011
TSH (uIU/mL, mean ± SD)	1.61 ± 2.21	1.19 ± 0.92	1.57 ± 1.29	2.12 ± 3.53	0.39
Hemodynamic measurements					
Systolic BP (mmHg, mean ± SD)	124.88 ± 21.32	124.02 ± 27.69	127.16 ± 14.61	123.29 ± 21.32	0.75
Diastolic BP (mmHg, mean ± SD)	72.96 ± 11.63	77.26 ± 12.23	71.59 ± 11.43	70.53 ± 10.55	0.064
Heart rate (bpm, mean ± SD)	84.52 ± 16.81	92.27 ± 16.63	84.74 ± 15.60	77.57 ± 15.61	0.004
Mean IVC diameter (mm)	15.7	12.6	15.5	18.9	0.003

BUN, blood urea nitrogen; BNP, brain natriuretic peptide; BP, blood pressure; BPM, beats per minute; Chemo, chemotherapy; IVC, inferior vena cava; SD, standard deviation; TSH, thyroid-stimulating hormone.

a Cardiac (e.g., hypertension), endocrine (e.g., adrenal insufficiency), pulmonary (e.g., chronic obstructive pulmonary disease), renal (chronic kidney disease), and gastrointestinal comorbidities were adjusted for in the analysis.

**Table 2:** Uni- and multivariate analyses of associations between undetectable BNP levels and small IVC diameter.

Univariate analysis			
Predictor variable	OR	95% CI	*p*-value
Small IVC diameter	3.92	1.55, 10.61	0.005
Multivariate analysis: Model 1			
Effect	OR	95% CI	*p*-value
Small IVC diameter	3.39	1.34, 9.27	0.01
Multivariate analysis: Model 2			
Effect	OR	95% CI	*p*-value
Small IVC diameter	7.04	2.31, 24.67	0.001

BNP, brain natriuretic peptide; CI, confidence interval; IVC, inferior vena cava; OR, odds ratio.

Univariate and multivariate analyses for associations between undetectable BNP and small IVC diameter.

Model 1: IVC diameter adjusted for age.

Model 2: IVC diameter adjusted for age, sex, BMI, systolic blood pressure, creatinine, and albumin.

## Discussion

As expected in our patients with cancer, BNP levels are positively correlated with overall IVC diameter. However, our study is the first to demonstrate a correlation between undetectable BNP (<10 pg/mL) and hypovolemic status assessed by IVC diameter, even after adjusting for relevant variables. This finding highlights the potential use of BNP levels as a non-invasive tool to assess volume status in this specific patient population. Nonetheless, while the wide CIs indicate limitations with relation to the small sample size, the association between undetectable BNP levels and hypovolemia, which were evaluated by IVC size, is supported by the large observed ORs. These large ORs are reassuring and suggest a potentially meaningful relationship that warrants further clinical investigation. If this relationship is confirmed in larger cohorts, this finding could have important implications for a non-invasive and cost-effective assessment of volume status in patients with cancer undergoing therapy. Second, the retrospective design of our study limits the ability to infer causality, as temporal relationships cannot be completely controlled. In particular, the temporal discrepancy between the BNP and the IVC measurements, which were performed within a 2-week window, may have introduced a variability and a biased component in the study, given the fluctuating nature of volume status. Third, we excluded patients with BMI > 30 kg/m^2^ to avoid a potential confounding factor, as obesity is known to suppress circulating BNP levels: prior community-based data demonstrated that obese individuals had significantly lower BNP concentrations compared with their lean counterparts (6).

However, excluding individuals with BMI >30 kg/m^2^ limits the generalizability of our findings in oncology populations. Further studies should include obese individuals to improve the real-world applicability of our study. Last, while BNP levels are frequently used to measure volume status, their gene expression can also be upregulated at a transcriptional and translational level by systemic inflammation and malignancy-related cytokines such as IL-6 and TNF-α in cardiac myocytes (7). This is relevant particularly in cancer patients, because BNP levels can be elevated in such patients independent of volume status. Although our study acknowledges this challenge in measuring BNP as a marker for cardiac volume status in cancer patients, we did not establish a control or measures for inflammatory markers. Overall, future research with larger sample sizes, wider demographics, and measurements taken closer together is recommended to further explore this relationship.

## Conclusion

In this study, it was found that in patients with cancer undergoing chemo- and/or radiation treatment, undetectable BNP levels were significantly associated with small IVC diameter as a marker of hypovolemia in multivariate analyses. In this group of patients, BNP measured with routine laboratory data is a readily available tool to identify individuals with hypovolemia where other measures of volume status such as physical examination findings may remain in the realm of uncertainty.

## Data Availability

The original contributions presented in the study are included in the article/Supplementary Material, and further inquiries can be directed to the corresponding author.
